# Choose, rate or squeeze: Comparison of economic value functions elicited by different behavioral tasks

**DOI:** 10.1371/journal.pcbi.1005848

**Published:** 2017-11-21

**Authors:** Alizée Lopez-Persem, Lionel Rigoux, Sacha Bourgeois-Gironde, Jean Daunizeau, Mathias Pessiglione

**Affiliations:** 1 Motivation, Brain and Behavior (MBB) team, Institut du Cerveau et de la Moelle épinière (ICM), Hôpital de la Pitié-Salpêtrière, Paris, France; 2 INSERM UMRS 1127, CNRS UMR 7225, Université Pierre et Marie Curie UPMC-Paris 6, Paris, France; 3 Max Planck Institute for Metabolism Research Cologne, Cologne, Germany; 4 Institute for Biomedical Engineering, University of Zürich and ETH Zürich, Translational Neuromodeling Unit, Zürich, Switzerland; 5 Laboratoire d'Économie Mathématique et de Microéconomie Appliquée (LEMMA), Université Panthéon-Assas, Paris, France; 6 Institut Jean-Nicod (IJN), CNRS UMR 8129, Ecole Normale Supérieure, Paris, France; Johns Hopkins University, UNITED STATES

## Abstract

A standard view in neuroeconomics is that to make a choice, an agent first assigns subjective values to available options, and then compares them to select the best. In choice tasks, these cardinal values are typically inferred from the preference expressed by subjects between options presented in pairs. Alternatively, cardinal values can be directly elicited by asking subjects to place a cursor on an analog scale (rating task) or to exert a force on a power grip (effort task). These tasks can vary in many respects: they can notably be more or less costly and consequential. Here, we compared the value functions elicited by choice, rating and effort tasks on options composed of two monetary amounts: one for the subject (gain) and one for a charity (donation). Bayesian model selection showed that despite important differences between the three tasks, they all elicited a same value function, with similar weighting of gain and donation, but variable concavity. Moreover, value functions elicited by the different tasks could predict choices with equivalent accuracy. Our finding therefore suggests that comparable value functions can account for various motivated behaviors, beyond economic choice. Nevertheless, we report slight differences in the computational efficiency of parameter estimation that may guide the design of future studies.

## Author summary

In economic decision theory, value is a construct that provides a metric to compare options: agents are likely to select options leading to high-value outcomes. In neuroscience, different behavioral tasks have been used to elicit the subjective values of potential outcomes, notably rating tasks, which demand an explicit value judgment on the outcome, and effort tasks, which demand an energetic expense (in order to increase outcome probability). However, it remains unclear whether the values elicited by these tasks are the same as the values that drive choices. Indeed, it has been argued that they involve different costs and consequences for the agent. Here, we compared value models that could account for behavioral responses in choice, rating and effort tasks involving the same set of options, which combined a monetary gain for the participant and a donation to a charity. We found that the most plausible model was that a same value function, with a similar selfishness parameter (relative weight on gain and donation), generated the responses in all three tasks. This finding strengthens the notion of value as a general explanation of motivated behaviors, beyond standard economic choice.

## Introduction

Value (or utility) functions have been defined to account for preferences revealed in choice tasks [1]. One basic principle is that if an agent prefers A over B, then for this agent the value of A is higher than the value of B. Assuming basic axioms of expected utility theory, cardinal functions have been described, such that option values can be positioned on a numeric scale [2]. Cardinal values rely on the notion that choice probability depends on the distance between option values, as well as on their distance from a reference point [3]. Value functions can be parameterized when choice options are combinations of objective quantities, e.g., the probability and magnitude of monetary payoff. The parameters can then be estimated through fitting procedures that maximize the likelihood of observed choices under the valuation model. Fitting choices involves specifying a function relating choice probability to option values, generally a *softmax* rule [4]. Thus, most studies have used choice data to infer functions that assign cardinal values to any possible option.

Alternatively, a more direct approach has been used in the neuroeconomics literature, using behavioral tasks in which subjects assign cardinal values to available options, instead of inferring value functions from their choices. One possibility is to ask subjects to rate on analog scale the desirability (or likeability) of the outcomes associated to the different options [5]. Another possibility is to ask subjects to express the maximal cost (e.g. price, effort or delay) that they are willing to endure in order to obtain these outcomes [6, 7]. The aim of the present study was to compare the value functions derived from these direct cardinal measures with the value functions derived from fitting choice data. We selected, in addition to a standard binary choice task where subjects state their preference between two options, a subjective rating task where subjects score the desirability of every possible outcome and an effort production task where the probability of obtaining the outcome depends on the force produced with a handgrip. Standard models of behavior in these tasks suggest that ratings and forces can be taken as direct measures of the subjective outcome values that drive choices (see Methods).

However, there are a priori reasons why the value functions elicited by the different tasks should differ in their form or in their parameters. In our perspective, the key difference between tasks is the nature of the cost. In choice tasks, the response entails an opportunity cost, corresponding to the value of the non-selected option [8]. The response is therefore based on the value difference between the two possible outcomes, which is often called decision value. As the motor response is generally similar for the two options, there is no need to consider action costs. In effort tasks, the response is associated with a specific cost due to energy expenditure, which may be signaled through muscular pain. The response therefore aims at maximizing the net value, i.e. the trade-off between outcome value and action cost [9]. In rating tasks, the variation in action cost across the possible positions on the scale is usually negligible, although the extremes may be longer to reach. Thus, the response should be a direct expression of outcome value. As decision values, net values and outcome values may be computed by different brain systems, they may follow different functions [10].

In addition, there is a cost that may be common to all behavioral tasks, which is social reprobation. Some responses may be more socially acceptable than others, particularly if moral considerations are involved [11]. This social cost may be more salient in rating tasks, which have no other consequences and can therefore be considered as ‘hypothetical’ decisions. By opposition, choice and effort tasks are typically consequential: they determine the outcome, either deterministically or probabilistically, and therefore involve ‘real’ decisions. Hypothetical and real decisions have been compared in a number of studies using various tasks [12–16], with contrasted results and no proper model comparison. Yet it may seem intuitive that subjects in rating tasks are more likely to pretend having values they do not have, for reputation concerns, because there is no obvious costly consequence. To assess this potential difference between tasks we used options that combined money for the subject (gain) and money for a charity (donation), with the aim of triggering moral dilemma.

Also, each behavioral task may be susceptible to specific artifacts. For instance, the rating scale is somewhat arbitrary, and may yield distortions of value functions due to framing or anchoring phenomena [17], particularly if subjects are not familiar with the range of values spanned in the set of options. Effort exertion, between zero and maximal force, may be less arbitrary but susceptible to fatigue, which may increase with the number of performed trials and influence effort cost, and hence the values expressed by participants [18].

In the present study, we compared the value functions elicited by the different tasks for a same set of composite outcomes, each combining gain and donation. We found that the same valuation model provide the best fit of behavior in the three tasks, with slight differences in parameter estimates.

## Results

### Model-free analysis of behavioral responses

**Fig 1:**
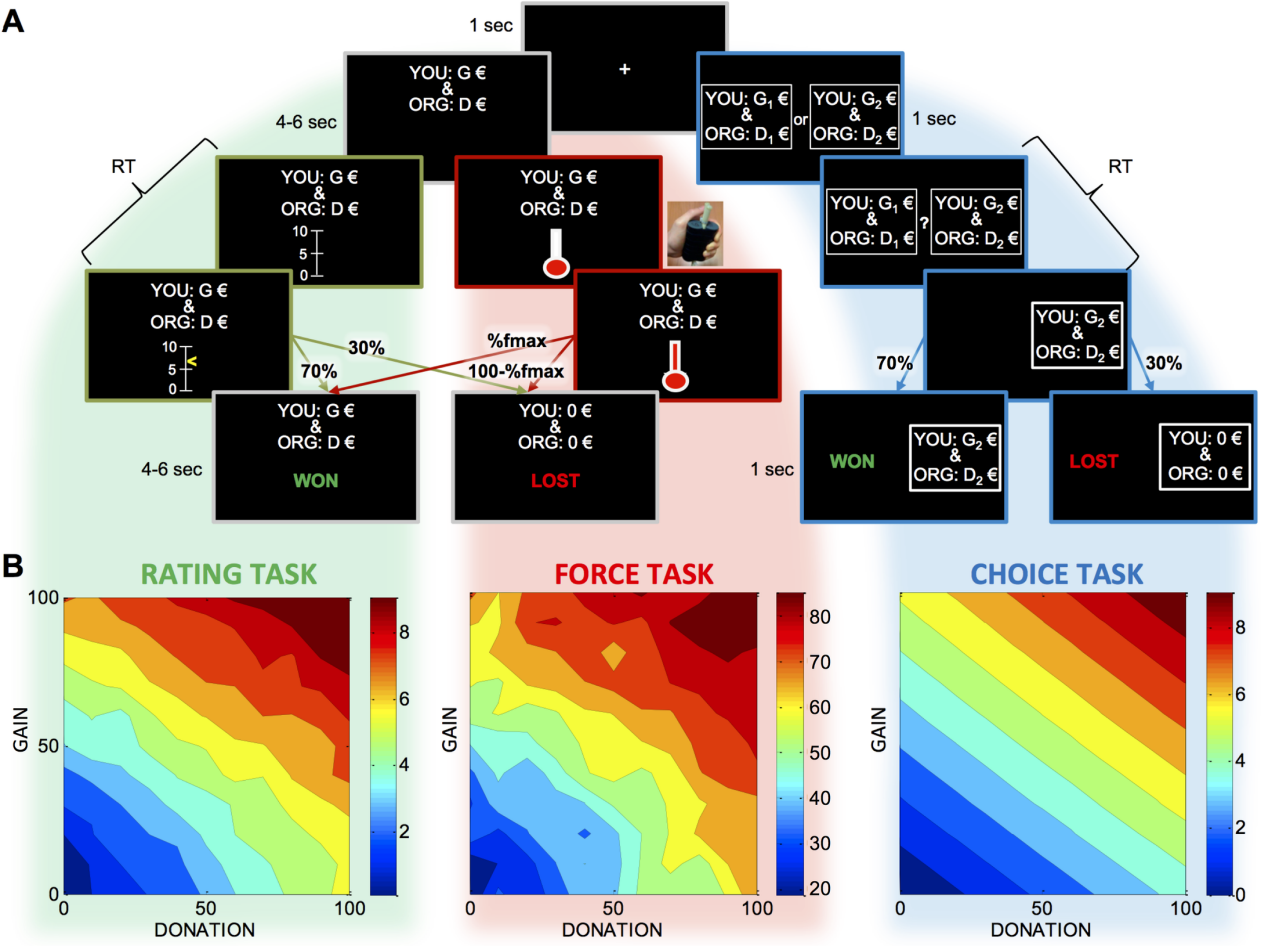
Task design and behavioral results. **A**. From top to bottom, successive screen shots of example trials are shown with their duration for the three tasks (left: rating task, middle: force task, right: choice task). Every trial started with a fixation cross. In the force and rating tasks, a single composite proposition, with a gain G for the subject (YOU) and donation D for the charity organization (ORG) was displayed on the screen. Then a scale (for rating) and a thermometer (for force) respectively appeared on the screen, noticing subjects that it was time for providing a response. After response completion (rating or force), feedback on whether the proposition was won or lost was displayed. The probability of winning was fixated to 70% in the rating task and determined by the percentage of maximal force produced in the force task. A loss meant no money for both the subject and the charity. In the choice task, two composite options were displayed and choice was triggered by switching ‘or’ into ‘?’. Feedback was winning the chosen option in 70% of the trials, and nothing in the remaining 30%. **B**. Average ratings (left), forces (right) and values inferred from choices (right) are shown as functions of the amount of gain and donation. Cold to hot colors indicate low to high values. The value function used to fit the choices was the a priori function that served to optimize the design (linear model with interaction).

Subjects (n = 19) participated in three tasks aimed at measuring subjective values of bi-dimensional outcomes composed of one gain for themselves and one donation for a charity organization they selected prior to the experiment (Fig 1, top). In the rating task, participants rated how much they would like to obtain the composite outcome using a scale graduated from 0 to 10. The feedback was probabilistic and they obtained the outcome in 70% of the trials, irrespective of their ratings, which were therefore not consequential. The probabilistic contingency was adjusted so as to match that of the effort task. In the force task, subjects had to squeeze a handgrip knowing that the chance to win the outcome was determined by the ratio of the force they produced during the trial and their maximal force measured beforehand. Note that previous experiments in the lab using the grip task with similar range of incentives showed that subjects produce on average about 70% of their maximal force [19]. In the choice task, participants had to choose between two composite options, the selected outcome being obtained in 70% of trials. The choice task followed an adaptive design [20] in which options were proposed so as to optimize the parameterization of an *a priori* value function (linear integration of gain and donation with their interaction).

As expected, explicit ratings, forces produced and subjective values inferred from choices all increased with incentives, i.e. with both gain and donation (Fig 1, bottom). Before going into more sophisticated models, we conducted linear regressions (for ratings and forces) or logistic regression (for choices) against the two main factors (gain G and donation D) and their interaction. Regression estimates obtained for main factors were significantly different from zero in all cases: in the rating task (β_R_(G) = 0.07±6.10^−3^, t(18) = 11.5, p = 1.10^−9^; β_R_(D) = 0.06±7.10^−3^, t(18) = 8.2, p = 1.10^−7^), in the force task (β_F_(G) = 0.05±6.10^−3^, t(18) = 8.5, p = 1.10^−7^; β_F_(G) = 0.05±6.10^−3^, t(18) = 7.2, p = 9.10^−7^) and in the choice task (β_C_(G) = 0.16±0.03, t(18) = 5.6, p = 2.10^−5^; β_C_(G) = 0.12±0.02, t(18) = 5.4, p = 4.10^−5^). Interaction terms were significant for the rating and force tasks but not for the choice task (β_R_(G*D) = -2.10–4±9.10^−5^, t(18) = -2.7, p = 0.01; β_F_(G*D) = -3.10–5±1.10^−5^, t(18) = -2.6, p = 0.02; β_C_(G*D) = 1.10–5±2.10^−4^, t(18) = 0.1, p = 0.95). In none of the tasks did we find a significant difference between the weights of gain and for donation, although there was a trend in favor of selfishness (R: t(18) = 1.79, p = 0.089; F: t(18) = 1.10, p = 0.29; C: t(18) = 1.70, p = 0.11).

We also regressed the residuals of this regression against trial and session number, in order to test for fatigue effects. As none of these tests was significant (all p>0.1), we did not include any parameter accounting for fatigue in our computational models. Finally, we compared the distribution of forces and ratings, irrespective of gain and donation. As uncertainty was controlled by force production in the effort task, the distribution could be affected by risk attitude, relatively to the rating task in which uncertainty was constant. Indeed, subjects should avoid medium forces, if they are risk averse, or on the contrary favor them, if they are risk seeking. We thus fitted a second-order polynomial function to individual distributions of forces and ratings. The coefficients of quadratic regressors were significant for both tasks (F: b = -0.31 ± 0.11, t(18) = -2.75 p = 0.013, R: b = -0.21 ± 0.06, t(18) = -3.34, p = 4.10–3), with no significant difference between tasks (t(18) = -0.85, p = 0.41). There was therefore no evidence that risk attitude created a difference between forces and ratings.

However, these model-free analyses do not provide any formal conclusion about how value functions differ across tasks, so we now turn to a model-based Bayesian data analysis.

### Bayesian comparison of valuation models

In order to further investigate how changing the elicitation paradigm could affect the subjective value of potential outcomes, we defined a set of twelve value functions that could explain the observed behavior in each task (see Methods). These value functions represent different ways of combining the two dimensions (gain and donation) composing the outcomes proposed in the tasks. They were used to generate forces and ratings with linear scaling (with slope and intercept parameters) and choices with logistic projection (*softmax* function with temperature parameter). All value functions were fitted on behavioral responses for every subject and task using Variational Bayesian Analysis (VBA) [21, 22]. The explained variance (averaged across subjects) was comprised between 43 and 70% in the force task, between 57 and 85% in the rating task and between 45 and 85% in the choice task. These results show that, for all three tasks, there were important differences in the quality of fit between value functions, which we compare below.

#### Comparison of value functions

**Fig 2:**
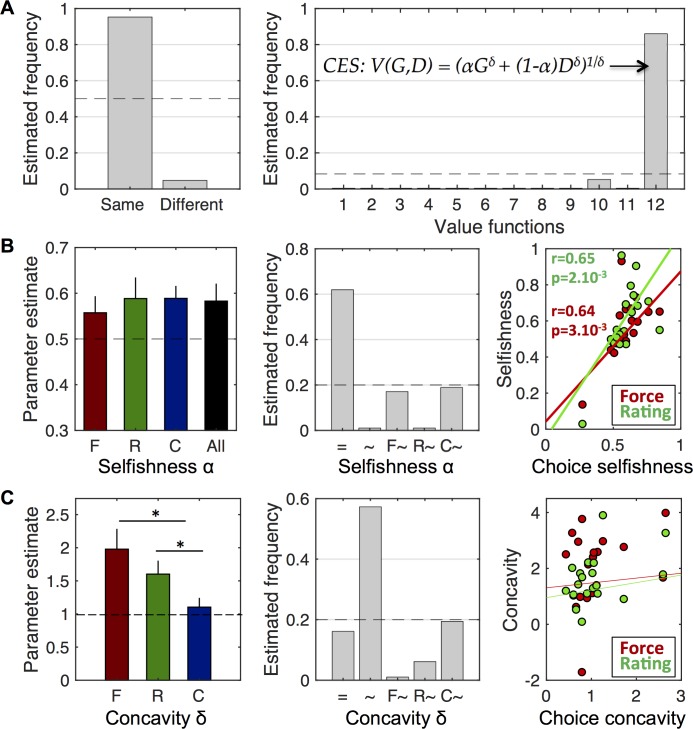
Comparison of value functions and their parameters. **A.** Comparison of value functions underlying behavior in the three tasks. Left: Estimated frequency for the family of models in which the three tasks are explained by the same value function and the family of models using different value functions. Right: Estimated frequencies obtained for the twelve models (value functions) belonging to the ‘same’ family. The winner is CES function (model 12), see equation on the graph, with V(G,D) the value of gain G and donation D, α the selfishness parameter and δ the concavity parameter. Dashed lines indicate chance levels (one over the number of models) **B.** Comparison of selfishness parameter across tasks. Left: Mean parameter estimates in the three tasks (F, R and C) separately and in the three tasks together (All). The dashed line (α = 0.5) indicates no bias toward one or the other dimension (gain or donation). Error bars indicates S.E.M. Middle: Estimated frequencies of models including one single selfishness parameter for the three tasks (=), three different selfishness parameters (~), or only one different from the two other (F~, R~, C~, with ‘X~’ standing for ‘task with a different parameter’). Dashed line indicates chance level. Right: Correlation of selfishness parameters between choice and force tasks (red) and between choice and rating tasks (green) across subjects. **C.** Same analysis as in B but for the concavity parameter. The dashed line in the left graph (δ = 1) corresponds to the linear model. Stars indicate significant differences between tasks.

First, using group-level Bayesian model comparison [22, 23], we examined whether behavior in the three tasks could be explained by the same value function. We found that the family of models with the same value function for the three tasks is far more plausible than the family of models with different value functions (Ef = 0.95, Xp = 1, Fig 2A). This indicates that there is no qualitative difference between the value functions underlying behavior in the three tasks. In turn, this enabled us to pool model evidences over the three tasks, and identify the most likely model (if any) for the common underlying value function.

Second, we found that the value function called ‘Constant Elasticity of Substitution’ (CES, see Methods) provides the best account of behavioral responses in the three tasks, as shown by the model comparison performed inside the ‘same’ family: Ef = 0.61, Xp = 1, Fig 2A). In what follows, we ask whether there are quantitative differences between value functions elicited by the three tasks, as could be captured by the CES fitted parameters.

#### Comparison of free parameters

The CES function is characterized by two main parameters: a "selfishness" parameter α comprised between 0 and 1 (α closer to 1 denotes more selfish behavior) and a concavity parameter δ (δ>1 indicates more sensitivity to high values in a composite proposition). We thus compared the fitted parameters of the CES model between tasks using ANOVA followed by t-tests. We only found a difference for the concavity parameter (F(2,54) = 3.72, p = 0.03; Fig 2C). More precisely, the concavity parameter in the choice task was significantly lower than in the two other tasks (δ_F_ = 1.98±0.31; δ_R_ = 1.60±0.20; δ_C_ = 1.10±0.14; δ_F_ vs δ_R_: t(19) = 1.55, p = 0.14; δ_F_ vs δ_C_: t(19) = 2.9, p = 9.10^−3^; δ_R_ vs δ_C_: t(19) = 2.56, p = 0.02). There was no significant difference in the selfishness parameter (F(2,54) = 0.09, p = 0.91; Fig 2B) which shows similar overweighting of gain relative to donation (α_C_ = 0.58±0.04, α_F_ = 0.56±0.05, α_R_ = 0.58±0.05). Nevertheless, since the absence of significance does not provide evidence for a null difference, we assessed this particular question with another Bayesian model comparison.

For each subject, we pooled the data acquired in the three tasks prior to fitting five distinct CES models: a model including one single selfishness parameter for all the tasks, a model including three different selfishness parameters, and all the intermediate variants (α_F_ = α_R_≠α_C_; α_F_ = α_C_≠α_R_; α_R_ = α_C_≠α_F_; see Methods). According to group-level Bayesian model comparison, the model with a unique selfishness parameter provided the best explanation to the pooled data (Ef = 0.52, Xp = 0.97, Fig 2B). We also found that the ensuing common selfishness parameter is significantly favoring the individualistic gain in the proposition (α = 0.58±0.04, t(19) = 2.16, p = 0.044). We also ran a similar analysis on the concavity parameter to assess whether the rating and force tasks could be explained by a unique parameter since the difference between them was not deemed significant. The winning model (Ef = 0.46, Xp = 0.92, Fig 2C) was the model with task-specific concavity parameters. This suggests that despite the absence of significant difference between δ_F_ and δ_R_ on average, the data are better explained with different parameter values.

Those results were confirmed by the significant correlations across subjects found in all pairs of tasks for the selfishness parameter (force and rating: r = 0.90, p = 1.10^−7^; force and choice: r = 0.64, p = 3.10^−3^; rating and choice: r = 0.65, p = 2.10^−3^), contrasting with the absence of significant correlation for the concavity parameter (Fig 2B and 2C, right panels). Moreover, we also compared the rankings on selfishness that the different tasks provided. We found significant correlations between all tasks taken two by two (force and rating: r = 0.80, p = 5.10^−5^; force and choice: r = 0.72, p = 5.10^−4^; rating and choice: r = 0.64, p = 3.10^−3^). Similar correlation coefficients and p-values were found with rankings of parameters, suggesting that the same subjects were identified as least or most selfish by the different tasks.

Taken together, these analyses allow us to conclude that the tasks used to access subjective values had an impact on the concavity of the value function but not on the weight given to the attributes.

#### Comparison of estimation efficiency

Finally, we asked which task actually provides the most efficient estimation of the underlying value function, if any.

**Fig 3:**
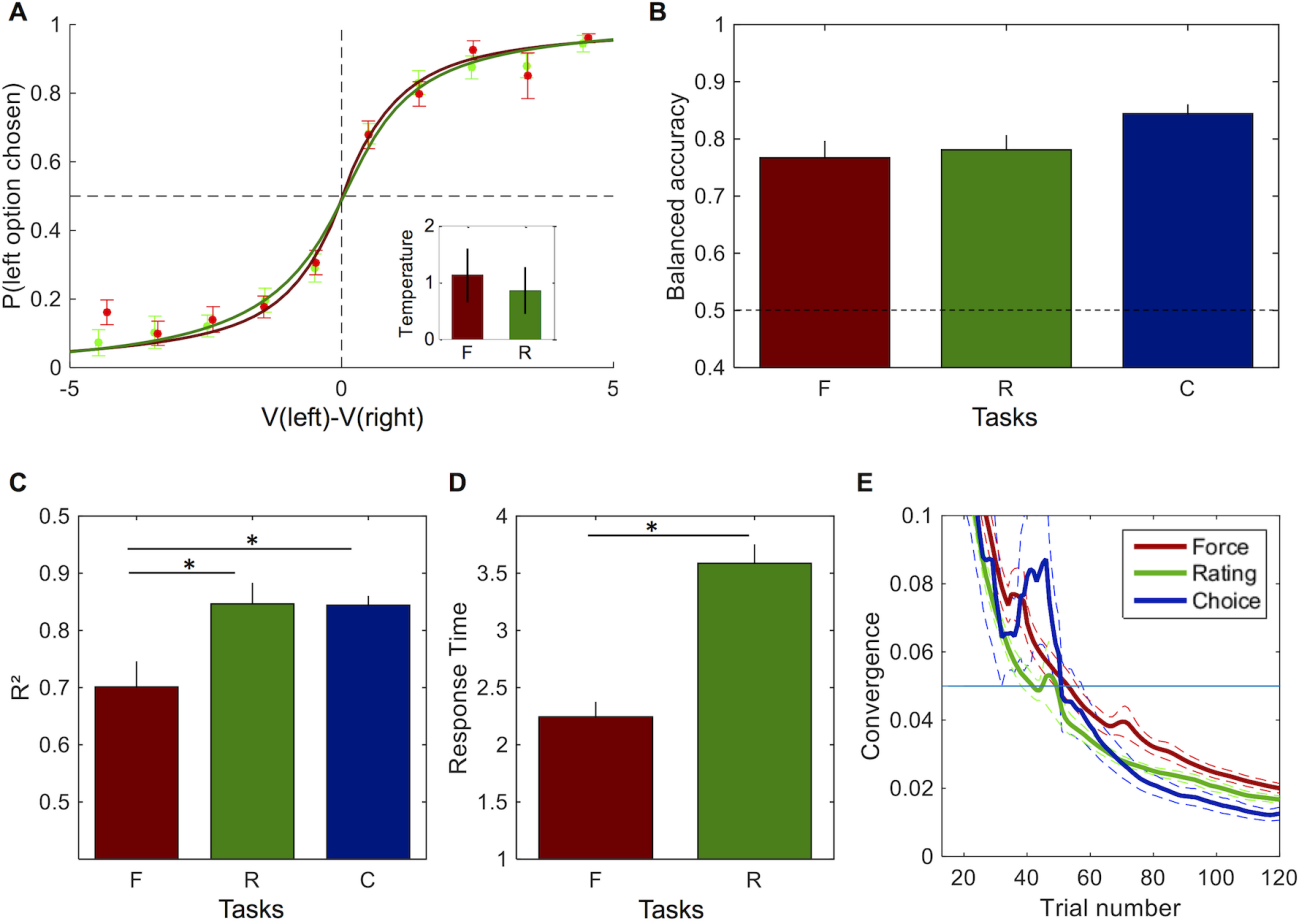
Comparison of estimation efficiency. **A**. Proportion of choices according to the difference between option values computed with the CES value function inferred either from the force task (red) or the rating task (green). Observed choices (circles) were fitted using logistic regression (continuous lines). Inset represents temperature estimates from logistic fits. **B**. Balanced accuracy according to the CES value function inferred from the force (red) and rating (green) tasks. **C**. Coefficient of determination R^2^ for the fit of each task (Force, Rating and Choice). **D**. Response time in force and rating tasks. **E**. Convergence measure according to trial number (with optimized trial order) in the force (red), rating (green) and choice (blue) tasks. Error bars indicate S.E.M. Stars indicate significant differences between two tasks.

To begin with, we assessed to what extent choices could be predicted from the other measures. Thus, we compared the balanced accuracy predicted by the values computed from the rating and the force tasks (same CES function with different selfishness and concavity parameters). We found no significant difference between them (t(19) = 0.82, p = 0.42), with balanced accuracy for each of them (Force: 77±3%; Rating: 78±2%) close to the balanced accuracy obtained with the value function inferred from choices (84±2%). Moreover, when fitting a logistic regression on choices with the rating and force values, we could not find any significant difference in the temperature parameter (β_F_ = 1.13±0.46; β_R_ = 0.86±0.40; t(19) = 1.36, p = 0.19). This suggests that rating and force measures were equally good to predict choices (Fig 3A and 3B).

Then, in order to further compare the efficiency of value estimation between tasks, we examined goodness-of-fit, task duration, and number of trials.

First, we compared the goodness-of-fit between tasks (Fig 3C). We found that the CES function provided a better fit for the rating and choice tasks compared to the force task (R^2^_F_ = 0.70±0.04; R^2^_R_ = 0.85±0.04; R^2^_C_ = 0.84±0.02; R^2^_F_ vs R^2^_R_: t(19) = -4.78, p = 1.10^−4^; R^2^_F_ vs R^2^_C_: t(19) = -3.87, p = 1.10^−3^; R^2^_c_ vs R^2^_R_: t(19) = -0.09, p = 0.93). There was no significant difference of goodness-of-fit between the choice and the rating task. This suggests that the force data were noisier than the two other measures.

Second, we compared the time needed to provide an answer in the rating and force tasks (Fig 3D). We did not include the choice task in this analysis because of the difference in the timing of options presentation. Moreover, the number of options to consider in the choice task is not the same, which would obviously bias the comparison. We found that response time in the force task was shorter than in the rating task (RT_F_ = 2.24±0.12 sec; RT_R_ = 3.59±0.16 sec; t(19) = 6.01; p = 1.10^−5^). Thus, given the same amount of trials, the force task was overall shorter to run than the rating task.

Third, we compared the number of trials needed in each task to yield efficient parameter estimates (Fig 3E). Recall that, in contradiction with the choice task, no adaptive design procedure was used for both force and rating tasks in our design (a fixed number of 121 options were presented in both cases). Nevertheless, one can derive a pseudo "convergence" measure for both tasks, in the aim of guessing what the amount of trials would have been, if one had used an adaptive design procedure. Note that the same approach can be taken post-hoc on the choice task, to yield a fair comparison. We thus derived such a convergence measure (see Methods) to determine the trial number after which the marginal gain was below our convergence criterion of 5%. This convergence measure was computed either on the sequence of trials as it unfolded during the experiment ("native order"), or by reordering the trials according to how informative they were ("optimized order"). Although the trend was for a reduction with the optimized order, we found no significant difference between the two estimations, neither in the rating task (native: 46±4 trials, optimized: 42±2 trials, difference: t(18) = 0.81, p = 0.43), nor in the force task, (native: 57±2trials, optimized: 57±3 trials, difference: t(18) = 0.08, p = 0.94) and choice task (native: 54±8 trials, optimized: 60±7 trials, difference: t(18) = 0.51, p = 0.62). With the optimized order, we found a significant difference of the pseudo-convergence trial number only between the rating and force tasks (γ_R_ vs γ_F_: t(18) = 5.57, p = 4.10^−5^; γ_R_ vs γ_C_: t(18) = 0.12, p = 0.9; γ_C_ vs γ_F_: t(18) = 1.75, p = 0.10). Without order optimization, there was no significant difference (all p>0.05). The trend was nonetheless that the force task required more trials than the rating task for converging on parameter estimation, as observed with the optimized order.

## Discussion

In this study, we showed that three tasks varying on several features elicited the same value function accounting for participants’ behavior. Moreover, the most critical parameter, precisely the relative weighting of gain and donation (selfishness), was similar in the three tasks. However, we found some differences in the concavity of value functions. In addition, the different tasks presented practical advantages and disadvantages that should be taken into account when selecting a particular elicitation procedure.

We showed with a Bayesian model comparison that the same value function could account for the three types of behavior. It is interesting to note that Bayesian inference enables concluding in favor of the null hypothesis, which cannot be formally validated from an absence of significant difference in classical statistical inference. The null hypothesis (no difference in value function) is consistent with subjects maximizing simple net utility functions defined as the difference between expected outcome values in the choice task, the expected outcome value minus a quadratic effort cost in the effort task, and the similarity of overt rating and covert judgment in the rating task (see Methods). This means that the computational processes used to generate the different behaviors (choice, rating, force) from underlying outcome values have no backward influence on these values. As a consequence, the results reported in the neuroeconomic literature using the different tasks, regarding the brain valuation system in particular, can be directly compared.

The winning value function, called ‘Constant Elasticity of Substitution’ [24], has been shown to provide a good account of choices made by participants in other experiments that involved sharing money with others [25], which is consistent with the present results. It has the advantage of simplicity, with only two parameters: one controlling the relative weighting of outcome dimensions (here, the selfishness parameter) and one controlling the interaction between dimensions (the concavity parameter). Note that the other value functions used in the model comparison also provided a satisfying fit of behavioral data, capturing the relative sensitivity to gain and donation. Thus, we do not wish to make a strong claim that the CES function should be used in any task assessing altruistic behavior. We simply used it in the following because it was the best candidate function to investigate the integration of outcome dimensions.

The three tasks not only shared the same value function, but also elicited similar selfishness parameters. Thus, the differences in the consequentiality of the behavioral response, and in the nature of associated costs, did not impact the effective weights assigned to the gain and donation dimensions. This may come as a surprise, given that exhibiting altruism comes for free (with no cost) in the rating task but not in the choice task (where there is an opportunity cost) or the force task (where there is an effort cost). This result suggests some stability across elicitation procedures in how dimensions are weighted. It is consistent with previous studies reporting similar values for hypothetical and real decisions [12–14]. In our data, the selfishness parameter denoted a preference for gain over donation, which is consistent with what has been observed in studies investigating altruism [26, 27]. Yet we note that our participants appeared less selfish, possibly because we asked them to select a NGO which they would give money to, instead of asking them to share money with another participant who they did not know.

We acknowledge that our demonstration of a same value function for different tasks suffers from some limitations. First, the range of costs involved in the choice and effort tasks remained reasonable. It is likely that costs should be integrated in the value function if they get more extreme (say if winning one euro for a charity demands days of work). Second, the stability of elicited value functions was assessed within subjects, which may favor consistency in behavioral responses. Results might have been more variable had we tested separate groups of subjects on the different tasks or the same subjects on different days. Indeed, the measures might be differentially sensitive to states such as mood or fatigue, which were not controlled in our design. Third, our conclusion could be specific to the particular dimensions that composed the outcomes presented in our tasks. Further experiments would be needed to generalize the result to other multi-attribute options, as in for example risky or inter-temporal choice, or to more natural multidimensional options such as food items.

Even if the same value function and the same selfishness parameter could explain the behavior in the three tasks, we found a significant difference between tasks in the concavity parameter. Indeed, the choice task did not reveal any concavity, indicating no interaction between dimensions, whereas the force task, and to a lesser extent the rating task, revealed a concavity, denoting a biased sensitivity to high monetary amounts, irrespective of the receiver. It remains difficult to conclude whether the concavity seen in rating and force tasks denotes an artifactual distortion of the actual value function or a better sensitivity to actual values, compared to the choice task which is more complex (with four numbers to be integrated). Indeed, concavity in the effort task may be higher because the effort cost function is not quadratic, as we assumed for the sake of simplicity. One may also speculate that high amounts trigger arousal responses, which may affect effort production but choice or rating. Alternatively, concavity in the choice task may be absent because in most cases, there are high amounts in both options. Note that choice options in our design were selected to optimize a value function (linear with interaction) where there was no concavity parameter. Nevertheless, even if no concavity was observed on average in the choice task, the model with a concavity parameter was favored by the Bayesian selection. This means that some subjects were better fitted with concave and others with convex value functions. This inter-subject variability possibly reflects differences in the sensitivity to equity (options with similar amount for them and for the charity).

Independently of the elicited value function, we assessed how the tasks differed in terms of precision and speed of parameter estimation. The choice and rating tasks were better fitted, with higher coefficients of determination than the force task. However, the value functions inferred from the rating and force tasks were equally capable of predicting choices. It was therefore not that the value function elicited with the force task was distorted or variable, but simply that the force data were noisier. Thus, if the objective is to predict choices, there is no reason, based on the accuracy criterion, to prefer any particular task.

On the other hand, response times recorded in the force task were shorter than in the rating task. Moreover, without design optimization, there was no significant reduction in the number of trials needed for stabilizing parameter estimation with the rating task compared to the force task. Thus, the speed criterion (total task duration) seems to be in favor of the force task. Note that this advantage could vanish if responses were mapped to ratings in a different way, for instance with one key per value. Also, the effort task requires some equipment and a calibration phase to determine maximal force, which may mitigate the gain in task duration.

Finally, for a similar precision and speed, the choice task needs an adaptive design (for the selection of choice options), which implies to posit priors on value functions and on parameters, whereas the other tasks can be run in a model-free manner. Thus, the simplest way to experimentally measure subjective value functions might not, eventually, be the binary choice task that is standard in behavioral economics.

### Conclusion

To our knowledge, this is the first study comparing direct elicitation of cardinal values (rating and force tasks) to ordinal rankings (choice task) for a same set of options. Those tasks are widely used in neuroeconomics and it is somewhat comforting that they reveal similar value functions driving the behavior despite trivial differences. They nonetheless present different advantages and drawbacks that may guide the design of future studies.

## Methods

### Ethics statement

The study was approved by the Pitié-Salpétrière Hospital ethics committee. All subjects were recruited via e-mail within an academic database and gave informed consent before participation in the study.

### Participants

Participants were right-handed, between 20 and 30 years old, with normal vision and no history of neurological or psychiatric disease. They were not informed during recruitment that the task was about giving money to a charity, in order to avoid a bias in the sample. Nineteen subjects (10 females; age, 22.2 ± 1.4) were included in the study. They believed that the money won while performing the task would be their remuneration for participating, but eventually, their payoff was rounded up to a fixed amount (100€).

### Behavioral tasks

Subjects performed the three tasks, the order being counterbalanced across subjects for the force and rating tasks. The choice task was always performed after the two others, which were performed during MRI scanning for other purposes.

The force task was preceded by maximal force measurement for the right hand [6]. Participants were verbally encouraged to squeeze continuously as hard as they could until a line growing in proportion to their force reached a target displayed on a computer screen. Maximal force was defined as the maximal level reached on three recordings. Then subjects were provided a real-time feedback about the force produced on the handgrip, which appeared as a red fluid level moving up and down within a thermometer, the maximal force being indicated as a horizontal bar at the top. Subjects were asked to try outreaching the bar and state whether it truly corresponded to their maximal force. If not, the calibration procedure was repeated.

In the force and rating tasks, 121 trials were presented in a random order across three sessions of 40 or 41 trials. Each trial corresponds to one of the 121 combinations of the experiment design (eleven possible incentives for themselves by eleven possible incentives for charity donation: from 0€ to 100€ with steps of 10€). Subjects performed the three sessions with the right hand, with short breaks between sessions to avoid muscle exhaustion.

In the force and rating tasks, each trial started by revealing the potential outcome, composed of two monetary incentives, with the inscriptions “YOU” followed by the amount for the subject, and “ORG” followed by the amount for the charity (Fig 1, top). The outcome was displayed for a duration jittered between 4 and 6 seconds. In the force task, subjects knew that the probability to win the outcome was proportional to the force they would produce after the display of the thermometer on the screen. More precisely, the probability of winning was equal to the percentage of their maximal force that they produced in the current trial. Subjects were also instructed to manage their forces in the effort task to avoid any frustration due to potential fatigue effect, and to use breaks between sessions to recover their muscular strength. During task trials, they were provided with online feedback on the exerted force (via a fluid level moving up and down within a thermometer). They were also informed that they had to produce a minimal effort in every trial (10% of their maximal force) and that the trial would be over when they stop squeezing the handgrip. Each trial ended with the display of the final outcome of their effort, for a duration jittered between 4 and 6 seconds, via the words “WON” (with the proposed monetary earnings) or “LOST” (with null earnings for both subject and charity).

The rating task only differed at the time of the motor response. Instead of a thermometer, a vertical rating scale from 0 to 10 units appeared after presentation of the potential outcome. Subjects were asked to rate the desirability of the outcome on the screen by moving the cursor through button presses with the right hand (index and middle finger for moving the cursor left and right, and ring finger for validating the response). They were asked to use the whole scale across trials. They were also informed that their rating would have no impact on the final outcome. They were then shown the final outcome that was randomized to obtain a “WON” in 70% trials, and a “LOST” 30% of trials (i.e., a proportion similar to that obtained in the force task).

The binary choice task included 200 trials, each presenting two composite options, one on each side of the screen. After considering the two options for 2 seconds, subjects could indicate the one they would prefer to win using their right hand (index vs. middle finger for left vs. right option). This option was actually won in 70% of trials, which was indicated with a positive feedback (“WON”) accompanied by the selected earnings. In the other 30% of trials, a negative feedback (“LOST”) was shown with a null outcome (0€) for both receivers.

Given the number of options in our design, there were 121^2^ (14641) possible binary choices. Constraints can be applied to reduce this number: choices are informative only if options are crossed (attributes never dominate on both dimensions), if options differ on both dimensions, and if the pair of options was not previously presented. However, those constraints only reduced the number of choices to 3025. Thus, we used an online optimization design to exploit the fact that some options are more informative than others to estimate a value function. At each trial, the design was optimized over a single dimension (gain or donation). The chosen combination was the one that minimized the trace of the posterior covariance matrix over the parameters of an a priori value function defined as follows: *V*(*G*,*D*) = *β*_*G*_ * *G* + *β*_*D*_ * *D* + *β*_*GD*_ * (*G* * *D*), corresponding to a linear integration with interaction [20]. Contrary to the force and rating tasks, the amounts for subjects and charity could vary with steps of 1€ (still between 0€ and 100€), since options were optimized for each trial and subject.

Subjects were informed that three trials would be randomly drawn (one per task) and that the average outcome would be actually implemented (including both their gain and donation). They were aware that their responses in the rating task would have no influence on the outcome, whereas they would have an impact in the effort and choice task. The uncertainty about winning the outcome was fixed to 70% in the choice and rating tasks, but controlled by the force produced in the effort task. As expected, the average forces were not significantly different from 70% (65±3%, p>0.1), and hence matched the uncertainty level of the other tasks.

### Data analysis

#### Model space

To investigate how the two attributes (gain G and donation D) were integrated into a subjective value, we compared 12 models with different value functions, based on behavioral data obtained in each task.

We first considered very simple models based on a single dimension, either the minimum value as in ‘mini’ (1), also called ‘Leontief utility’ [25], or the maximum value as in ‘maxi’ (2).

Mini:V(G,D)=min⁡(αG,βD) (1)

Maxi:V(G,D)=max⁡(αG,βD) (2)

The six following models are based on Park and colleagues’ study [28]. They were initially used to examine the integration of positive and negative values into an overall subjective value, which we extrapolated to the integration of money received and money allocated (to a charity). These models differ on the presence of an interaction between attributes and on the presence of a non-linear transformation of attributes, which should be concave for gains and convex for losses, according to prospect theory [3]. In addition, the non-linear transformation could be similar or not (same parameter or not) for gains and losses. We refer to these models as (3) linear—independent, (4) similarly nonlinear—independent, (5) nonlinear—independent, (6) linear—interactive, (7) similarly nonlinear—interactive and (8) nonlinear—interactive.

Linear-independent:V(G,D)=αG+βD (3)

$$Similarly\;non\text{-}linear\;\text{-}\;independent:\;V(G,D)=\alpha G^{\delta }+\beta D^{\delta }$$ (4)

$$Non\text{-}linear\;\text{-}\;independent:\;V(G,D)=\alpha G^{\delta }+\beta D^{\varepsilon }$$ (5)

Linear-interactive:V(G,D)=αG+βD+γGD (6)

$$Similarly\;non\text{-}linear\;\text{-}\;interactive:\;V(G,D)=\alpha G^{\delta }+\beta D^{\delta }+\gamma G^{\delta }D^{\delta }$$ (7)

$$Non\text{-}linear\;\text{-}\;interactive:\;V(G,D)=\alpha G^{\delta }+\beta D^{\varepsilon }+\gamma G^{\delta }D^{\varepsilon }$$ (8)

In order to complete those six models, we have included other standard value functions used in previous studies. Notably, some models have been developed to account for the potential intrinsic value of equity, as suggested by equity theory [29]. For instance, the model that we have called linear—equity (9) integrates a proxy for inequity: the absolute difference between gain and donation.

Linear-equity:V(G,D)=αG+βD+γ|G−D| (9)

Another function has been proposed by Fehr and Schmidt to explain inequity aversion [30], a model (10) that we also included.

Fehr&Schmidt:V(G,D)=⁡G−αmax⁡(D−G,0)−βmax⁡(G−D,0) (10)

Finally, we have included production functions, even if they were not developed in the context of altruistic donation, because they implement other ways of combining two dimensions. The simplest is the Cobb-Douglas production function (11), which is both multiplicative and non-linear.

$$Cobb\text{-}Douglas:\;V(G,D)=G^{\delta }*D^{1-\delta }$$ (11)

A more general form is the CES function (12), commonly used to account for consumer behavior [25], with a parameter *α* for linear weighting of dimensions and a parameter *δ* for concavity of preferences. Note that Leontief, Linear and Cobb-Douglas functions are special cases of the CES function.

$$Constant\;Elasticity\;of\;Substitution\;(CES):\;V(G,D)={\left(\alpha G^{\delta }+(1-\alpha )D^{\delta }\right)}^{1/\delta }$$ (12)

#### Value-response mapping

To formalize the link between behavioral responses and outcome values, we defined net utility functions in the three tasks.

In the choice task, responses are consequential because subjects can only win the chosen outcome (with 70% probability). In other words, the benefit associated to the choice is the expected value of the outcome (value times probability). The value of the unchosen outcome can be seen as an opportunity cost. Therefore, subjects should maximize the following net utility function:

$$U\;(C,G,D)\;=\;V_{C^{1}}(G,D)*0.7-V_{C^{0}}(G,D)*0.7\;=\;0.7*(V_{C^{1}}(G,D)-V_{C^{0}}(G,D))$$ (13)

With *C*^1^ and *C*^0^ being chosen and unchosen, respectively. Thus, choice rate should scale to the distance between outcome values. This distance is classically transformed into choice probability through a *softmax* function [4]. For the probability of choosing the left option the *softmax* function is:

$$P(G_{L},D_{L})\;=\;\frac{1}{1+e^{-\frac{V(G_{L},D_{L})-V(G_{R},D_{R})}{\beta }}}$$ (14)

With V(*G*_*L*_,*D*_*L*_) and V(*G*_*R*_,*D*_*R*_) the values of left and right options, and *β* the temperature (choice stochasticity). Obviously the probability of selecting the other (right) option is:

$$P(G_{R},D_{R})\;=\;1-P(G_{L},D_{L})$$ (15)

In the rating task, responses are not consequential, since feedbacks (winning or not the outcome) are randomly drawn (with 70% probability). The reason why ratings are informative about values can only be that subjects wish to comply with instructions, and report their genuine judgment about outcome desirability. In other words, they try to minimize the error between overt ratings and covert judgments. Following a previously published model [31], they should maximize a net utility function defined as:

$$U(R,G,D)\;=-{\left(R-V(G,D)\right)}^{2}$$ (16)

With R being the potential rating. The optimal rating is the one that maximizes the net utility function, which quite trivially is just the outcome value:

argmax_RU=V(G,D) (17)

Thus, although we ignore the scale on which internal judgments are made, ratings should linearly reflect outcome values. Note that we neglect here the cost of moving the cursor along the scale, which could favor medium ratings. This might shrink the rating distribution but not alter the linear scaling.

In the effort task, responses are consequential, since the force produced determines the probability of winning the outcome. In addition, this task also entails an effort cost, which is modeled as a supralinear function of force in motor control theory [9]. Thus, a simple net utility function (see [32] for a recent use) that subjects should maximize inludes a quadratic effort cost that is subtracted from the expected outcome value (value times probability):

$$U\;=\;V(G,D)*F-\gamma *F^{2}$$ (18)

With F being the potential force (and outcome probability), and *γ* a parameter scaling effort cost to expected value. The force F* maximizing the net utility is:

$$argmax\_F\;U\;=\;F^{*}\;=\;\frac{V(G,D)}{2\gamma }$$ (19)

Therefore, forces should linearly reflect outcome values. Note that the uncertainty component (controlled by F) cancels out and observed forces can be used as direct readouts of subjective values. We also neglected the cost of time here. Although it is true that producing higher forces takes more time (at the scale of ms), this could only change the scaling between forces and values, not the linear relationship.

Responses were modeled with a linear function for both the rating and effort tasks:

R(G,D)=aV(G,D)+b (20)

With R the rating assigned to a potential outcome composed of gain G and donation D, scaled by parameters a and b. The same linear function was used to generate forces, with different scaling parameters a and b.

#### Model fitting and comparison

Every model was fitted at the individual level to ratings, forces and choices using the Matlab VBA-toolbox (available at http://mbb-team.github.io/VBA-toolbox/), which implements Variational Bayesian analysis under the Laplace approximation [21, 33]. This iterative algorithm provides a free-energy approximation to the marginal likelihood or model evidence, which represents a natural trade-off between model accuracy (goodness of fit) and complexity (degrees of freedom) [34, 35]. Additionally, the algorithm provides an estimate of the posterior density over the model free parameters, starting with Gaussian priors. Individual log-model evidences were then taken to group-level random-effect Bayesian model selection (RFX-BMS) procedure [22, 23]. RFX-BMS provides an exceedance probability (Xp) that measures how likely it is that a given model (or family of models) is more frequently implemented, relative to all the others considered in the model space, in the population from which participants were drawn [22, 23].

The first model comparison was done to determine whether the same value function was used across the three tasks. For this purpose, 12^3^ = 1728 models were built with every possible combination of functions across tasks. We then calculated the model evidence for the models that included the same function for all tasks (‘same’ family) and for all the other models (‘different’ family), following the procedure proposed by [22]. We then used family-wise inference at the group level to estimate the probability that participants used the same value function in the different tasks [36].

The second model comparison was done to assess whether the same selfishness and concavity parameters (in the winning CES value function) could be used in the three tasks. For this purpose 5 models were built for each parameter, representing all possible combinations:

- ρ_F_≠ρ_R_≠ρ_C_
- ρ_F_ = ρ_R_≠ρ_C_
- ρ_F_≠ρ_R_ = ρ_C_
- ρ_F_ = ρ_C_≠ρ_R_
- ρ_F_ = ρ_R_ = ρ_C_

#### Convergence assessment

In order to assess convergence of model fitting, we estimated the parameters of the CES function iteratively, including trials one at a time. At each step we calculated the increase in estimation precision γ:

$$\gamma_{t}\;=\;\frac{\sigma_{t-1}-\sigma_{t}}{\sigma_{t-1}}$$ (21)

with *σ*_*t*_ the mean posterior variance (over all parameters) at trial t. This convergence measure tracks the information gain afforded by each trial. The convergence threshold was set at 5%, i.e. the minimum number of trials was defined as the last trial in which the convergence measure was above 5%. As the convergence measure was monitored separately for the three tasks, the minimal number of trials needed to reach the threshold can be used to compare their efficiency in eliciting the parameters of the CES function. This convergence measure can be derived post-hoc using either the native or the optimized sequence of trials. For the latter, the first eleven trials were chosen so as to cover the range of possible gains and donations (with amounts of 0, 30, 50, 70 and 100€ in both dimension), in a randomized order. Then, to optimize information gain, the next options were selected at each trial such that the trace of the expected posterior matrix would be minimized.

## References

[1] Samuelson PA. The numerical representation of ordered classifications and the concept of utility. *Rev Econ Stud* 1938; 6: 65–70.

[2] Von Neumann J, Morgenstern O. Theory of games and economic behavior. Princet Univ Prress Princet.

[3] Kahneman D, Tversky A. Prospect theory: An analysis of decision under risk. *Econom J Econom Soc* 1979; 263–291.

[4] Luce RD. On the possible psychophysical laws. *Psychol Rev* 1959; 66: 81 13645853 10.1037/h0043178

[5] Lebreton M, Jorge S, Michel V, Thirion B, Pessiglione M. An Automatic Valuation System in the Human Brain: Evidence from Functional Neuroimaging. *Neuron* 2009; 64: 431–439. doi: 10.1016/j.neuron.2009.09.040 19914190 10.1016/j.neuron.2009.09.040

[6] Pessiglione M, Schmidt L, Draganski B, Kalisch R, Lau H, Dolan RJ, et al How the Brain Translates Money into Force: A Neuroimaging Study of Subliminal Motivation. *Science* 2007; 316: 904–906. doi: 10.1126/science.1140459 17431137 10.1126/science.1140459 PMC2631941

[7] Plassmann H, O’Doherty J, Rangel A. Orbitofrontal Cortex Encodes Willingness to Pay in Everyday Economic Transactions. *J Neurosci* 2007; 27: 9984–9988. doi: 10.1523/JNEUROSCI.2131-07.2007 17855612 10.1523/JNEUROSCI.2131-07.2007 PMC6672655

[8] Posnet J, Ian S. Indirect cost in economic evaluation: the opportunity cost of unpaid inputs. *Health Econ* 1996; 5: 13–23. doi: 10.1002/(SICI)1099-1050(199601)5:1<13::AID-HEC182>3.0.CO;2-J 8653189 10.1002/(SICI)1099-1050(199601)5:1<13::AID-HEC182>3.0.CO;2-J

[9] Rigoux L, Guigon E. A Model of Reward- and Effort-Based Optimal Decision Making and Motor Control. *PLoS Comput Biol* 2012; 8: e1002716 doi: 10.1371/journal.pcbi.1002716 23055916 10.1371/journal.pcbi.1002716 PMC3464194

[10] Rangel A, Camerer C, Montague PR. A framework for studying the neurobiology of value-based decision making. *Nat Rev Neurosci* 2008; 9: 545–556. doi: 10.1038/nrn2357 18545266 10.1038/nrn2357 PMC4332708

[11] Polnaszek TJ, Stephens DW. Why not lie? Costs enforce honesty in an experimental signalling game. *Proc R Soc B Biol Sci* 2013; 281: 20132457–20132457. 10.1098/rspb.2013.2457 PMC3843836 24225460

[12] Kühberger A, Schulte-Mecklenbeck M, Perner J. Framing decisions: Hypothetical and real. *Organ Behav Hum Decis Process* 2002; 89: 1162–1175.

[13] Bickel WK, Pitcock JA, Yi R, Angtuaco EJC. Congruence of BOLD Response across Intertemporal Choice Conditions: Fictive and Real Money Gains and Losses. *J Neurosci* 2009; 29: 8839–8846. doi: 10.1523/JNEUROSCI.5319-08.2009 19587291 10.1523/JNEUROSCI.5319-08.2009 PMC2749994

[14] Schmidt L, Palminteri S, Lafargue G, Pessiglione M. Splitting Motivation: Unilateral Effects of Subliminal Incentives. *Psychol Sci* 2010; 21: 977–983. doi: 10.1177/0956797610372636 20511391 10.1177/0956797610372636

[15] Kang MJ, Rangel A, Camus M, Camerer CF. Hypothetical and Real Choice Differentially Activate Common Valuation Areas. *J Neurosci* 2011; 31: 461–468. doi: 10.1523/JNEUROSCI.1583-10.2011 21228156 10.1523/JNEUROSCI.1583-10.2011 PMC6623437

[16] FeldmanHall O, Dalgleish T, Thompson R, Evans D, Schweizer S, Mobbs D. Differential neural circuitry and self-interest in real vs hypothetical moral decisions. *Soc Cogn Affect Neurosci* 2012; 7: 743–751. doi: 10.1093/scan/nss069 22711879 10.1093/scan/nss069 PMC3475363

[17] Lynch JG, Chakravarti D, Mitra A. Contrast Effects in Consumer Judgments: Changes in Mental Representations or in the Anchoring of Rating Scales? *J Consum Res* 1991; 18: 284.

[18] Le Bouc R, Rigoux L, Schmidt L, Degos B, Welter M-L, Vidailhet M, et al Computational Dissection of Dopamine Motor and Motivational Functions in Humans. *J Neurosci* 2016; 36: 6623–6633. doi: 10.1523/JNEUROSCI.3078-15.2016 27335396 10.1523/JNEUROSCI.3078-15.2016 PMC6601748

[19] Schmidt L, d’Arc BF, Lafargue G, Galanaud D, Czernecki V, Grabli D, et al Disconnecting force from money: effects of basal ganglia damage on incentive motivation. *Brain* 2008; 131: 1303–1310. doi: 10.1093/brain/awn045 18344560 10.1093/brain/awn045

[20] Daunizeau J, Preuschoff K, Friston K, Stephan K. Optimizing Experimental Design for Comparing Models of Brain Function. *PLOS Comput Biol* 2011; 7: e1002280 doi: 10.1371/journal.pcbi.1002280 22125485 10.1371/journal.pcbi.1002280 PMC3219623

[21] Daunizeau J, Friston KJ, Kiebel SJ. Variational Bayesian identification and prediction of stochastic nonlinear dynamic causal models. *Phys Nonlinear Phenom* 2009; 238: 2089–2118. 10.1016/j.physd.2009.08.002 PMC2767160 19862351

[22] Rigoux L, Stephan KE, Friston KJ, Daunizeau J. Bayesian model selection for group studies—Revisited. *NeuroImage* 2014; 84: 971–985. doi: 10.1016/j.neuroimage.2013.08.065 24018303 10.1016/j.neuroimage.2013.08.065

[23] Stephan KE, Penny WD, Daunizeau J, Moran RJ, Friston KJ. Bayesian model selection for group studies. *NeuroImage* 2009; 46: 1004–1017. doi: 10.1016/j.neuroimage.2009.03.025 19306932 10.1016/j.neuroimage.2009.03.025 PMC2703732

[24] Armington PS. A Theory of Demand for Products Distinguished by Place of Production. *Staff Pap* 1969; 16: 159–178.

[25] Andreoni J, Miller J. Giving according to GARP: An experimental test of the consistency of preferences for altruism. *Econometrica* 2003; 70: 737–753.

[26] Hutcherson CA, Bushong B, Rangel A. A Neurocomputational Model of Altruistic Choice and Its Implications. *Neuron* 2015; 87: 451–462. doi: 10.1016/j.neuron.2015.06.031 26182424 10.1016/j.neuron.2015.06.031 PMC4947370

[27] Varnum MEW, Shi Z, Chen A, Qiu J, Han S. When ‘Your’ reward is the same as ‘My’ reward: Self-construal priming shifts neural responses to own vs. friends’ rewards. *NeuroImage* 2014; 87: 164–169. doi: 10.1016/j.neuroimage.2013.10.042 24185022 10.1016/j.neuroimage.2013.10.042

[28] Park SQ, Kahnt T, Rieskamp J, Heekeren HR. Neurobiology of Value Integration: When Value Impacts Valuation. *J Neurosci* 2011; 31: 9307–9314. doi: 10.1523/JNEUROSCI.4973-10.2011 21697380 10.1523/JNEUROSCI.4973-10.2011 PMC6623498

[29] Adams JS. Inequity in social exchange. *Adv Exp Soc Psychol*; 2.

[30] Fehr E, Schmidt KM. A theory of fairness, competition, and cooperation. *Q J Econ* 1999; 114: 817–868.

[31] Lebreton M, Abitbol R, Daunizeau J, Pessiglione M. Automatic integration of confidence in the brain valuation signal. *Nat Neurosci* 2015; 18: 1159–1167. doi: 10.1038/nn.4064 26192748 10.1038/nn.4064

[32] Lockwood PL, Hamonet M, Zhang SH, Ratnavel A, Salmony FU, Husain M, et al Prosocial apathy for helping others when effort is required. *Nat Hum Behav* 2017; 1: 131. 10.1038/s41562-017-0131 PMC5555390 28819649

[33] Daunizeau J, Adam V, Rigoux L. VBA: A Probabilistic Treatment of Nonlinear Models for Neurobiological and Behavioural Data. *PLoS Comput Biol* 2014; 10: e1003441 doi: 10.1371/journal.pcbi.1003441 24465198 10.1371/journal.pcbi.1003441 PMC3900378

[34] Friston K, Mattout J, Trujillo-Barreto N, Ashburner J, Penny W. Variational free energy and the Laplace approximation. *NeuroImage* 2007; 34: 220–234. doi: 10.1016/j.neuroimage.2006.08.035 17055746 10.1016/j.neuroimage.2006.08.035

[35] Penny WD. Comparing dynamic causal models using AIC, BIC and free energy. *Neuroimage* 2012; 59: 319–330. doi: 10.1016/j.neuroimage.2011.07.039 21864690 10.1016/j.neuroimage.2011.07.039 PMC3200437

[36] Penny WD, Stephan KE, Daunizeau J, Rosa MJ, Friston KJ, Schofield TM, et al Comparing Families of Dynamic Causal Models. *PLoS Comput Biol* 2010; 6: e1000709 doi: 10.1371/journal.pcbi.1000709 20300649 10.1371/journal.pcbi.1000709 PMC2837394

